# Evaluation of isometric strength and fatty infiltration of the subscapularis in latarjet surgery

**DOI:** 10.1590/1413-785220152303144944

**Published:** 2015

**Authors:** Ricardo Barreto Monteiro dos Santos, Fábio Neumann Kauffman, Gabriel Praxedes de Lima, Avraham Machado Costa Ferreira, Saulo Monteiro dos Santos, José Lamartine de Andrade Aguiar

**Affiliations:** 1 Universidade Federal de Pernambuco, Recife, PE, Brazil; 2 Hospital Miguel Arraes, Recife, PE, Brazil

**Keywords:** Shoulder dislocation, Muscle strength, Orthopedics

## Abstract

**OBJECTIVE:**

To evaluate the function of the subscapularis muscle by means of isometric strength, clinical examination and analysis of fatty infiltration in patients with recurrent anterior dislocation of the shoulder undergoing Latarjet-Patte surgery.

**METHODS:**

38 patients operated from March 2011 to March 2012, with minimum follow-up of two years were evaluated, being 26 males and 12 females, with a mean age of 28.7 years old. Isometric strength was measured using a portable dynamometer and measuring the distance from the back of the hand during the lift-off test. We used the Rowe and Walch-Duplay scores for clinical evaluation. The degree of fatty infiltration of the subscapularis belly was assessed by computed tomography.

**RESULTS:**

The mean scores in the Walch-Duplay and Rowe were 84.7 and 89.4, respectively. The mean distance to the back of the hand was 7.34 cm on the operated side and 8.72 cm on the opposite side (p <0.0001). The mean strength measured in the lift-off test was 0.38 kg lower than on the contralateral side (p = 0.001). There was no fatty infiltration of the subscapularis in 16 patients (42.1%). Sixteen patients (42.1%) were classified as Goutallier grade 1 and six (15.8%) as grade 2. We found that the measured isometric strength decreases with increasing the degree of fatty infiltration (p <0.0001).

**CONCLUSIONS:**

The decrease in subscapularis strength, albeit of low magnitude (0.38 kg), was directly related to the degree of fatty infiltration and worse clinical outcomes. *Level of Evidence III, Therapeutic Study - Investigating the Results of Treatment.*

## INTRODUCTION

The subscapularis muscle (SCM) is the main medial rotator of the humerus and contributes to the anterior dynamic stability of the glenoumeral joint.^1^ In surgical procedures for the treatment of anterior shoulder instability, the access route is through this important muscle, which can cause damage to its function.

The transfer of the coracoid process to the anterior edge of the glenoid, described by Latarjet^2^ in 1954, has shown good results in the treatment of anterior shoulder instability. Over time, however, this procedure was modified, in order to improve the stabilization mechanism and cause lesser damage to the subscapularis tendon.

The main mechanism of Latarjet stabilizer surgery is the tendinomuscular dynamic effect provided by the subscapularis together with the tendon. This dynamic effect promotes greater stability when compared to the Bankart procedure.^3^ Therefore, the Latarjet surgery is an alternative to the arthroscopic Bankart procedure, especially in individuals who practice contact and throwing sports.^4^^-^ 6

For access to the anterior border of the glenoid, the original Latarjet technique consists of tenotomy with detachment of the subscapularis. However, for the purpose of preserving the SCM tendon insertion, Walch^5^ popularized the Didier- Patte modified approach that uses longitudinal divulsion of the SCM, thereby maintaining the continuity of its muscle fibers. Therefore, this modification of the SCM approach is currently known in the literature as Latarjet-Patte surgery.

The objective of this study was to evaluate the function of the subscapularis muscle through isometric strength, the clinical examination and analysis of fatty infiltration by computed tomography in patients with recurrent anterior dislocation of the shoulder undergoing Latarjet-Patte surgery.

## MATERIALS AND METHODS

Between March 2011 and March 2012, 43 patients underwent surgery for the treatment of recurrent anterior shoulder dislocation by Latarjet-Patte technique at Clinical Hospital of the Federal University of Pernambuco and at Miguel Arraes Hospital. All patients had a previous history of recurrent traumatic dislocation of the shoulder. Two patients with bilateral instability and three patients with incomplete follow-up were excluded from this study, leaving 38 patients for the study. Twenty-six patients were male and 12 female, with a mean age of 28.7 years old (range 17-41 years old). Of the 38 patients evaluated, 16 (42.1%) practiced sports (football, volleyball and martial arts). The dominant side was affected in 24 patients (63.2%). (Table 1)

**Table 1.** Data from patients submitted to Latarjet-Patte procedure and control group (n=38).

Variable	Patients operated	Control Group
Patients (n)	38	30
Age (years old)		
Mean	28.7	28.9
Min	17	17
Max	41	40
Standard deviation	7.16	5.49
Gender n (%)		
Masculine	26 (68.4%)	20 (70%)
Feminine	12 (31.6%)	10 (30%)
Dominant side n (%)		
Right shoulder	36 (94.7%)	29 (96.7%)
Left shoulder	2 (5.3%)	1 (3.3%)
Affected side n (%)		
Right shoulder	26 (68.4%)	-
Left shoulder	12 (31.6%)	-
Physical activity n (%)		
Yes	21 (55.3%)	11 (36.7%)
Number of dislocations or sub-dislocations (number of events)		
Mean	5.15	-
Minimum	2	-
Maximum	14	-

Source: Data from this study.

Before surgery, all patients were evaluated by magnetic resonance imaging (MRI) and x-ray of the affected shoulder (true AP and axillary Bernageau profile). The blurring of the anterior edge of the glenoid was visible on radiographs in eight patients (21.1%) and was observed glenohumeral arthrosis grade 1, according to the classification proposed by Samilson and Prieto^7^ in three patients (7.9%). The MRI showed no tear of the subscapularis preoperatively and in all patients the Bankart lesion on the glenoid lip was observed. Follow-up time ranged from 24 to 39 months (average 28.6 months).

The study was approved by the Ethics Committees of both institutions involved and all patients agreed to participate in the study through the Free and Informed Consent.

## SURGICAL TECHNIQUE

The Latarjet-Patte surgery was performed in all patients by the main author. This procedure was performed according to the method described by Godinho and Monteiro^4 ^and Young *et al.* 8 with the patient positioned in "beach chair" by the deltopectoral approach, the cephalic vein was identified and laterally removed with the deltoid muscle. Next, the coracoid process was visualized and osteotomy was performed at the level of its curvature that was on average 2.5 cm in length. The coracoacromial ligament was identified and released in its acromial insertion, its full origin remaining in the coracoid. The obtained bone graft was inferiorly decorticated and then two 3.2 mm diameter holes were drilled along its central axis, preserving 1 cm distance between the holes. The glenoid approach was performed by divulsion in the longitudinal direction of the muscle fibers at the junction between the upper 2/3 and lower 1/3 of the subscapularis, aiming to preserve the motor innervation of the muscle, which was made in the upper 2/3 through the upper branch of the subscapular nerve and, in the lower 1/3, by the inferior branch of the same nerve. The coracoid process graft was fixed with two malleolar 4.5 mm screws in the anterior edge of the glenoid, below the equator, about 1 to 2 mm medial to the articular margin. The coracoacromial ligament was sutured to anterior capsule keeping at this time the upper limb at 90° abduction and maximum external rotation, so that stability was achieved without loss of range of motion and without risk of graft fracture. Then, the subcutaneous and skin were sutured to the deltopectoral fascia. The patient was immobilized with a Velpeau sling and discharge scheduled for the day after surgery.

Pendulum exercises were started from the 2^nd^ day after surgery. From the 21^st^ day began the rehabilitation protocol for shoulder instability designed by specialized physiotherapists. The return to sports activities, including contact sports, was allowed after the fourth month after surgery, when presenting bone healing of the glenoid graft in Bernageau profile radiography.

Patients were asked to attend the clinic after the 24^th^ month of surgery when it was evaluated the amplitude of the movements of the shoulders and evidence of sufficiency were held with lift-off test for the subscapularis. The results were assessed through Rowe^9^ and Walch-Duplay scores.^10^

**Figure 1.**
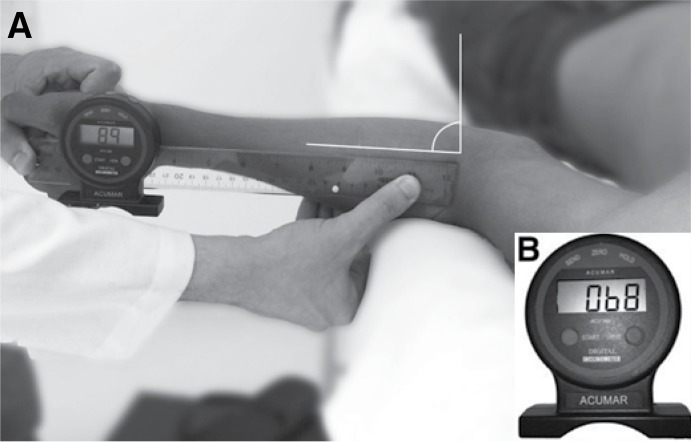
(A) Measurement of the external rotation amplitude with his elbow next to the body using a digital goniometer. (B) Detail of the apparatus used.

The lift-off test was performed by placing the back of the hand at the L3 level when the subscapularis is in its maximum contraction amplitude. Patients with subscapularis insufficiency were unable to withdraw the back of the hand from their back. This test was performed bilaterally and the data of the distance from hand to the back and isometric strength were collected. In order to measure strength, we used a portable dynamometer (model 01163, Lafayette Instrument Company, Lafayette, IN, USA), pre-programmed to measure for 5 seconds and record the peak in kg. (Figure 1) A second measurement was performed after 3 min of rest and the average of the two values was recorded. The contralateral side was also evaluated using the same methodology and the averages were compared using the Student *t*-test.

A control group of 30 volunteer participants (20 men and 10 women) asymptomatic for shoulder conditions and with demographics similar to the study group was used to assess whether there was strength difference in the lift-off test on the dominant side and not dominant one. Isometric strength was measured using the same methodology employed in the group of operated patients.

**Figure 2.**
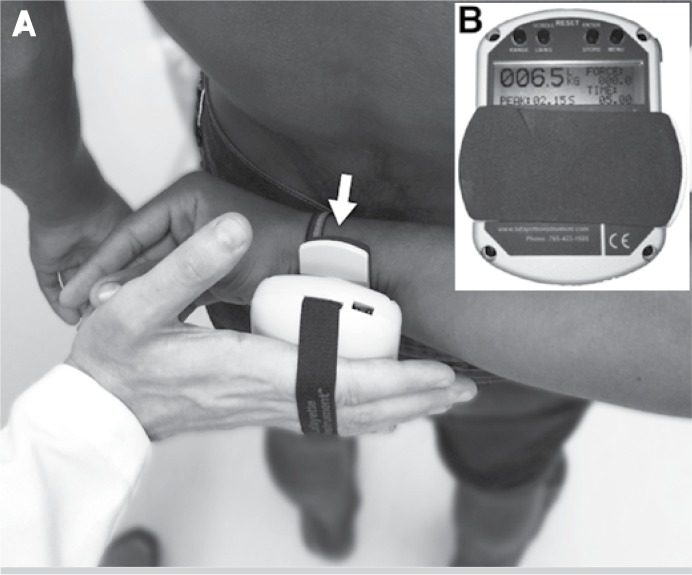
(A) Measurement of isometric strength during the lift-off test. (B) Detail of the apparatus used.

The range of motion was measured bilaterally using a digital goniometer (Acumar model AC002, Lafayette Instrument Company, Lafayette, IN , USA) using the methodology recommended by the European Society of Shoulder and Elbow. (Figure 2) CT scan was performed from the 24^th^ month after surgery. The protocol included 2mm slices with axial and sagittal reconstruction algorithms for soft tissue and bone window in order to evaluate the fatty infiltration of the SCM. Studies were interpreted by specialized radiologists in musculoskeletal system of the Clinical Hospital of the Federal University of Pernambuco and without knowledge of the clinical results. The degree of fatty infiltration was classified using the methodological criteria described by Goutallier* et al.* 11

For the descriptive statistics analysis average, minimum and maximum values and standard deviation were used. For analytical statistics of continuous variables, we used the nonparametric Mann-Whitney test, and the *t* test for independent variables. For variance analysis we used Tukey's post-test. We considered significant variables when p <0.05. The statistical calculation was performed with the capabilities of SPSS, version 22. 

## RESULTS

The control group consisted of 30 participants. The average strength measured in the dominant limb was 6.65kg and 6.53kg in the non-dominant. There was no statistically significant difference from the dominant and non-dominant hand (p=0.964). (Table 2)

**Table 2.** Assessment of strength of the control group at the lift-off test (n=30).

Variable	Mean (min - max)	p
Dominant side (kg)	6.65 (5.8 - 7.6)	} p=0.964*
Non dominant side (kg)	6.53 (5.5 - 7.3)	

T-Test for independent variables Source: Data from this study.

In the operated group, the mean score obtained in the Duplay score was 84.7 points, with excellent and good results in 31 patients (81.6%). The average Rowe score was 89.4 points, with satisfactory results in 30 patients (78.9%). There was only one case of recurrence of the dislocation, after two years of surgery, assigned to a new trauma. Three patients (7.9%) had persistent symptoms of anterior prehension, even after rigorous physical therapy rehabilitation, and in these cases we noticed on the radiograph that the bone graft was misplaced, that is, above the equator of the glenoid. (Table 3)

**Table 3.** Postoperative outcomes evaluated through Rowe and Walch- -Duplay scores. (n=38).

Scores evaluated	Mean (min - max)
Rowe score	
Stability (0-50 points)	46.3 (30-50)
Mobility (0-20)	15.7 (0-20)
Function (0-30)	27.3 (10-30)
Total	89.4 (45-100)
Walch-Duplay score	
Activities of daily living (0-25)	23.1(15-25)
Stability (0-25)	23.1 (15-25)
Pain (0-25)	17.6 (15-25)
Mobility (0-25)	20.7 (15-25)
Total	84.7 (60-100)

Source: dados from this study.

Regarding the range of motion, the mean lateral rotation with the elbow besides the body was 53.2º (range 10º to 80º) on the operated side and 63.4º (range 45º to 90º) in the non-operated side. The average deficit of lateral rotation on the operated side was 10° in relation to the non-operated side. In two cases we found difference higher than 30º between the operated and the non-operated side. We also observed on the operated sideZ an average limit of 10 degrees (0º to 30º) with the limb in lateral rotation and the arm in 90º abduction. The medial rotation was symmetrical in 32 patients (84.6%). (Table 4)

**Table 4.** Clinical outcomes of patients submitted to the Latarjet- -Patte procedure

Amplitude of movement	Operated shoulder	Contralateral shoulder	p
Amplitude of movement			
Lateral rotation with the elbow besides the body. Med (min. - max.)	53.2˚ (10˚-80˚)	63.4˚ (45˚-90˚)	<0.0001*
Lateral rotation with the abducted elbow at 90˚. Med (min. - max.)	82.1˚ (60˚-100˚)	92.2˚ (80˚-120˚)	<0.0001*
Medial Rotation (Vertebral levels) n (%)			
T4	26 (68.4%)	31 (81.6%)	
T6	1 (2.6%)	1 (2.6%)	
T7	10 (26.3%)	6 (15.8%)	
T9	1 (2.6%)	-	
Mean distance from hand to back on the lift-off test (cm)	7.34 (±0.50)	8.72(±0.24)	<0.0001^†^
Mean Isometric Strength at lift-off test (kg)	6.39 (±0.57)	6.77 (±0.35)	0.001^†^

* Mann-Whitney test.

† T-Test for independent variables. Source: dados from this study.

The average distance from hand to back in the lift-off test was 7.34 cm in the operated side and 8.72 on the opposite side (p <0.0001). The average strength measured by the lift-off test was 0.38kg smaller than the non-operated side (p=0.001). (Table 4)

**Figure 3.**
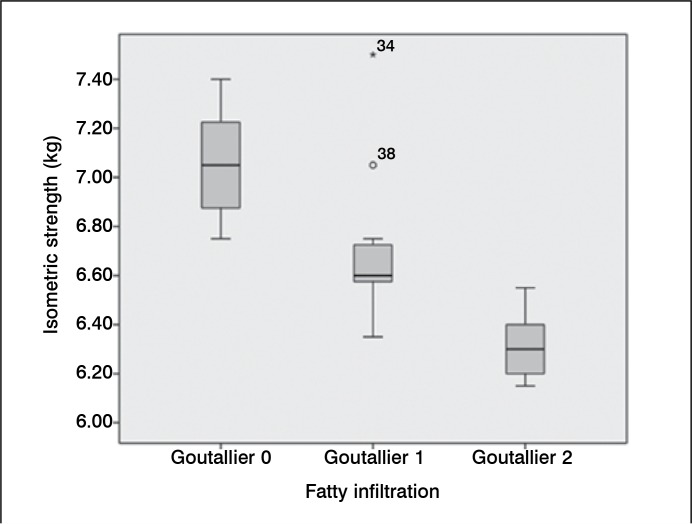
Values of isometric strength (kg) measured during the lift-off test correlated to the degree of fatty infiltration (Goutallier).

**Figure 4.**
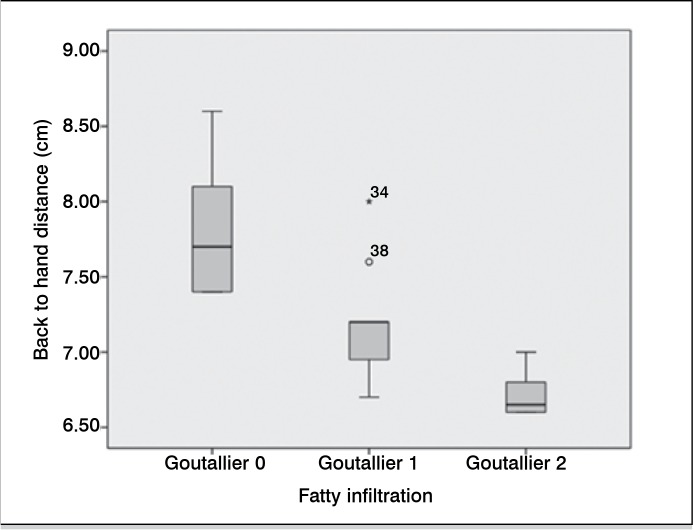
Measurement of the back to hand distance (cm) obtained during the lift-off test correlated with the degree of fatty infiltration (Goutallier).

In the tomographic evaluation of the SCM, at the end of the follow-up there was no fatty infiltration in 16 patients (42.1%). Sixteen patients (42.1%) were classified as grade 1 Goutallier and six patients (15.8%) as grade 2 Goutallier. We found that the measured isometric strength decreases with increasing the degree of fatty infiltration (p <0.0001). (Figure 3) On the other hand, we also found that the distance of hand to back measured in the lift-off test was inversely proportional to the degree of fatty infiltration, with statistical significance (p <0.0001). (Figure 4) Finally, we found that the average Rowe and Walch-Duplay scores were lower the higher the degree of fatty infiltration of the SCM. (Table 5)

**Table 5.** Functional outcomes stratified by fat degeneration (n=38).

Variable	Fatty infiltration (Goutallier)			p
	Grau 0	Grau 1	Grau 2	
Frequency n (%)	16 (42.1%)	16 (42.1%)	6 (15.8%)	
Mean isometric strength (kg)	7.05 (±0.2)*	6.67 (±0.27)*	6.31 (±0.14)*	<0.0001^†^
Distance hand to back (cm)	7.76 (±0.38)*	7.15 (±0.30)*	6.71 (±0.16)*	<0.0001^†^
Rowe score (0 - 100 points)	97.18 (±4.06)*	89.37 (±6.80)*	69.16 (±15.30)*	<0.0001^†^
Walch-Duplay score (0 - 100 points)	94.37 (±8.13)*	80.0 (±9.66)*	71.66 (±11.69)*	<0.0001^†^

† ANOVA. Turkey post-test;

* Statistically significant. Source: dados from this study.

## DISCUSSION

There is still controversy on the best method of treatment for recurrent anterior dislocation of the shoulder. Currently, the arthroscopic treatment of Bankart and the bone block procedure have been used. Among the advantages of the locking bone surgery using the Latarjet-Patte technique, we emphasize its superior stabilizing effect as compared to the arthroscopic Bankart surgery, being, therefore, better indicated for patients with high risk of recurrence, especially those who participate in contact sports.

Most authors agree that SCM dysfunction is associated with low levels of patient satisfaction. Studies by Sachs et al. found that in 30 patients undergoing Bankart open surgery 23% had incompetent SCM after four years follow-up. These authors concluded that only the SCM dysfunction was significantly correlated with bad outcomes.^12 ^Moreover, Scheibel et al.^13 ^assessed the previous shoulder stabilization by "L" shaped tenotomy of the SCM. These authors reported clinical signs of subscapularis insufficiency in 53.8% of patients and showed in MRI muscle atrophy and fatty infiltration, particularly of the upper belly of the subscapularis muscle. In agreement with these studies, Picard et al.^14^ also evaluated the anatomical and functional effects of the vertical approach of the subscapularis muscle during Latarjet procedure, after finding, following surgery, a loss of 50% strength and 50% of the thickness of the SCM.

Comparing the results after the Latarjet surgery using two forms of the SCM approaches, namely "L" shaped tenotomy and longitudinal divulsion in the longitudinal direction of the muscle fibers, Maynou et al.^15^ observed better clinical outcomes and lower fatty degeneration in patients operated with the longitudinal divulsion. They also found that the average muscle power in the lift-off test was also higher, being of 6.7kg in patients with SCM longitudinal approach and 4.8 kg with the SCM "L" -shaped incision. Elkousy et al.^16^ evaluated 30 patients undergoing Latarjet surgery using the longitudinal divulsion approach of the SCM fibers. These authors found a decrease of 0.3kg in the operated in relation to the non-operated during the belly press test, but this difference was not statistically significant.

Although it was not the aim of this study to compare the various SCM approach techniques, we found that there was reduced range of motion, especially the lateral rotation when compared to the non-operated side. Moreover, we found that the reduction of subscapularis strength, though of low magnitude (0.38kg), was directly related to the degree of fatty infiltration and worse clinical outcomes. 

During the preparation of this work we found some limitations in our study. First, we did not evaluate the EMG activation patterns as well as the contributions of the other muscles involved in medial rotation strength. Second, in the tomographic evaluation of fatty infiltration of the SCM, we did not evaluate which belly muscle of MSC was the most affected. Thirdly, although Rowe and Walch-Duplay scores are the clinical evaluation tools most commonly used, they cannot be considered the optimal measurement for the assessment of strength and subscapularis function. Although our study has identified the occurrence of dysfunction and fatty infiltration of the SCM through the longitudinal approach, it was not possible to identify, in our research, which were the factors that led to this dysfunction.

## CONCLUSION

We conclude that the results of this study confirm the need for integrity of the subscapularis tendon to maintain the medial rotation strength and shoulder function. Despite the good and excellent results in Rowe and Walch-Duplay scores, we believe it is necessary to consider the possibility of partial loss of SCM strength and function in patients undergoing Latarjet-Patte surgery, even if using the longitudinal divulsion of its fibers.
